# Synthesis and Performance Evaluation of Polyamine Boron Crosslinker for Gel Fracturing Fluid

**DOI:** 10.3390/gels12030236

**Published:** 2026-03-12

**Authors:** Quande Wang, Tengfei Dong, Qi Feng, Shengming Huang, Xuanrui Zhang, Guancheng Jiang

**Affiliations:** 1 College of Petroleum Engineering, China University of Petroleum (Beijing), 18 Fuxue Road, Changping, Beijing 102249, China; 15664628289@163.com (Q.W.); 18563073299@163.com (Q.F.); smhuang2015@163.com (S.H.); m15600263100_1@163.com (G.J.); 2 College of Science, China University of Petroleum (Beijing), 18 Fuxue Road, Changping, Beijing 102249, China; zxrscreaming@sina.com

**Keywords:** fracturing fluid, crosslinker, response surface methodology, dynamic reversible crosslinking

## Abstract

The fracturing development of low-permeability and ultra-low-permeability oil and gas reservoirs urgently requires a fracturing fluid that combines high performance and low damage. To overcome this challenge, this study synthesized a novel polyamine boron crosslinker (PBC) suitable for 0.2% guar gum. The molecular structure was characterized by Fourier transform infrared spectroscopy (FT-IR) and nuclear magnetic resonance hydrogen spectroscopy (^1^H NMR). Meanwhile, this study introduced the response surface methodology and established a second-order regression model to determine the optimal synthesis conditions (polyetheramine 10.8 g, n-butanol 7.4 g, and ethylene glycol 20.7 g) with a model prediction error of only 0.7%. The results indicated that PBC exhibited excellent performance in 0.2% guar gum. The viscosity of crosslinked gel fracturing fluid remained stable at approximately 100 mPa·s under 60 °C and 100 s^−1^ shear. The wall forming filtration coefficient was 2.30 × 10^−4^ m/s^1/2^, and the initial filtration was 1.30 × 10^−3^ m^3^/m^2^. The static settling rate was 2.4 cm·min^−1^, demonstrating good suspended sand capacity. Furthermore, the synergistic interaction between borate ester bond and polyetheramine in the PBC conferred dynamic reversible crosslinking and uniform network formation. This enabled high-strength, low-damage crosslinking effects at low concentrations. This study provides an efficient crosslinker solution for 0.2% guar gum, holding both theoretical and engineering significance for advancing the low-cost development of fracturing fluid.

## 1. Introduction

With the exploration and development of oil and gas resources, the reserves of medium- to high-permeability reservoirs are gradually depleted [1,2]. Consequently, the development of low-permeability reservoirs has become increasingly critical to meet global energy demands [3,4]. Hydraulic fracturing technology remains the core engineering approach for achieving the economical and efficient development of low-permeability and ultra-low-permeability reservoirs such as shale oil and tight gas [5]. The effectiveness of this technology highly relies on the performance of the fracturing fluid system. In water-based fracturing fluids, water-based gel fracturing fluids with guar gum and its derivatives as thickeners have comprehensive advantages such as low cost, easy preparation, and excellent sand-carrying performance, and occupy a dominant position in domestic and foreign oilfield sites [6]. However, traditional guar gum fracturing fluid systems typically require the use of higher concentrations of guar gum (0.3–0.6%) to ensure sufficient viscosity and sand-carrying capacity [7,8]. This not only significantly increases the material cost of fracturing operations, but also leads to an increase in the amount of insoluble residue left after gel breaking, causing reservoir damage to micro cracks and pore channels [9,10]. This seriously affects the ability of post compression drainage and the long-term flow capacity of cracks [11]. Therefore, it is necessary to develop a new fracturing fluid system with low concentration, high performance, and low damage. This will be an important direction for achieving cost reduction, efficiency improvement, and green sustainable development in the current field of oil and gas field production and transformation.

The key technology to reduce the dosage of guar gum lies in the development of efficient crosslinkers [12,13]. The crosslinker forms a three-dimensional network structure by coordinating or complexing with the adjacent cis hydroxyl groups on the guar gum molecular chain, thereby converting low-viscosity linear guar gum solution into high-viscosity elastic gel [14,15]. Among various types of crosslinkers, organic boron crosslinkers have outstanding advantages such as mild crosslinking conditions, controllable delayed crosslinking properties, good gel shear recovery, and thorough gel breaking [16,17]. These advantages make them the preferred choice for fracturing fluid systems in low-to-medium-temperature reservoirs (typically below 120 °C). In recent years, many researchers have improved the temperature resistance and crosslinking efficiency of boron crosslinkers by optimizing the structure of organic ligands (such as using polyols, sugars, alcohol amines, etc.), achieving a reduction in guar gum concentration while maintaining system performance [18,19].

Nevertheless, most existing organic boron crosslinkers still face significant challenges when applied in systems with guar hydrochloride concentrations below 0.25% [20,21]. The primary issue is insufficient crosslinking network strength, leading to a rapid decline in gel viscosity retention under high-temperature and high-shear conditions [22,23]. Second, they exhibit strong dependence on system pH, making performance stability highly susceptible to fluctuations in on-site water quality [24]. Furthermore, traditional synthesis processes are often based on single-factor empirical optimization, lacking systematic understanding and precise control over the complex interplay between key reactant ratios, reaction conditions, and the final product’s crosslinking behavior [25,26]. This limits further breakthroughs in performance and the expansion of application boundaries.

To address these challenges, this study presents a molecularly designed polyamine boron crosslinker (PBC) for ultra-low concentration guar gum systems. The central hypothesis is that incorporating polyetheramine segments—which contain both flexible ether linkages and amino groups—into an organoboron crosslinker structure can achieve a synergistic combination of dynamic borate ester bonding and enhanced polymer–crosslinker interactions. Specifically, the polyetheramine component is expected to serve multiple molecular-level functions: (1) The ether bonds provide enhanced chain flexibility and water solubility, enabling the formation of a more uniform and extensible three-dimensional network. (2) The amino groups can participate in hydrogen bonding with guar gum hydroxyl groups, potentially reinforcing the crosslinked structure without forming permanent covalent bonds. (3) The relatively long and flexible polyetheramine chains may act as molecular spacers, modulating crosslinking density and network architecture to achieve optimal performance at low guar gum concentrations [27]. The target product was synthesized in a one-step process using boric acid, polyetheramine, ethylene glycol, and n-butanol as raw materials. The molecular structure of the target product was characterized by FT-IR and ^1^H NMR. Unlike conventional organic boron crosslinkers, which typically consist of borate esters with simple polyols or alkanolamines, the PBC system integrates a polymeric amine component with multiple functional groups along a flexible backbone, representing a distinct molecular architecture. To overcome the limitations of traditional optimization methods, this study employed response surface methodology to systematically model and optimize key synthesis parameters, including the dosages of polyetheramine, ethylene glycol, and n-butanol. Furthermore, the comprehensive performance of the crosslinked gel fracturing fluid was systematically evaluated in terms of temperature and shear resistance, static filtration, sand-carrying capacity, and residue content after gel breaking, with comparative analysis against commercially available crosslinkers. This study not only aims to develop a novel crosslinker with superior performance but also seeks to elucidate the structure–activity relationships governing its behavior. By articulating a clear chemical hypothesis and differentiating our molecular design from previously reported systems, this work provides new theoretical insights and practical approaches for the rational design, controlled synthesis, and performance optimization of highly efficient organic boron crosslinkers for low-concentration guar gum fracturing fluids.

## 2. Results and Discussion

### 2.1. FT-IR

**Figure 1:**
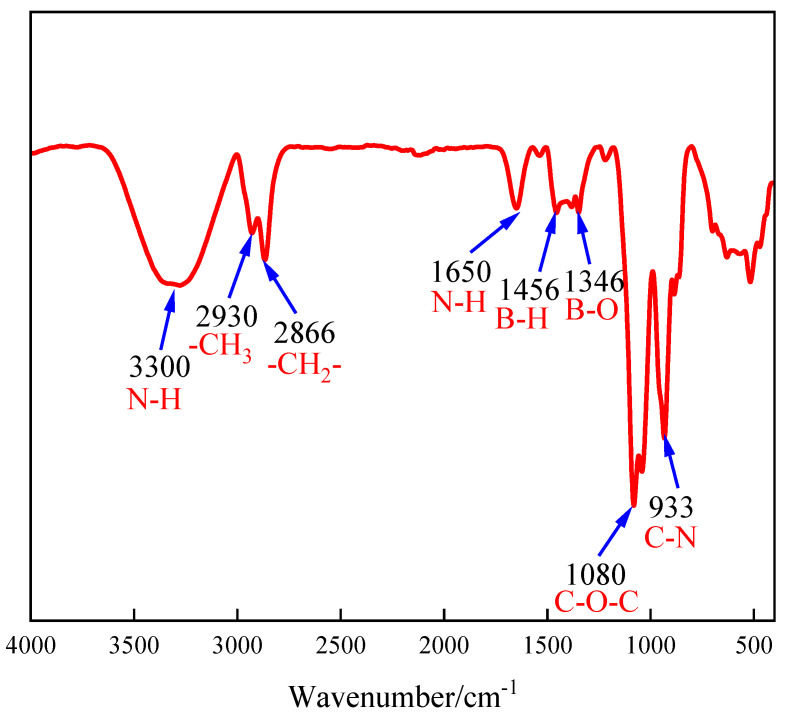
The FT-IR of PBC.

The characteristic functional groups of PBC were characterized by FT-IR, as shown in Figure 1. It can be seen from Figure 1 that the stretching vibration absorption peak of the N-H bond was located at 3300 cm^−1^. The absorption peaks of asymmetric and symmetric stretching vibrations of C-H bonds were located at 2930 cm^−1^ and 2866 cm^−1^, confirming the presence of methyl and methylene groups in the molecular structure. The bending vibration absorption peak of N-H bond was located at 1650 cm^−1^. At 1456 cm^−1^, there was an absorption peak for the bending vibration of the C-H bond or the stretching vibration of the B-N bond, which may overlap in this region. The stretching vibration absorption peak of the B-O bond was located at 1346 cm^−1^. The 1080 cm^−1^ was the asymmetric stretching vibration absorption peak of C-O-C ether bond. The 933 cm^−1^ was the stretching vibration absorption peak of the C-N bond, which may shift due to the presence of cyclic ether bonds and boronic ester rings in the molecule [28]. These spectral features collectively confirm that the molecular structure of the synthetic product is consistent with the designed polyamine boron crosslinker.

### 2.2. ^1^H NMR

**Figure 2:**
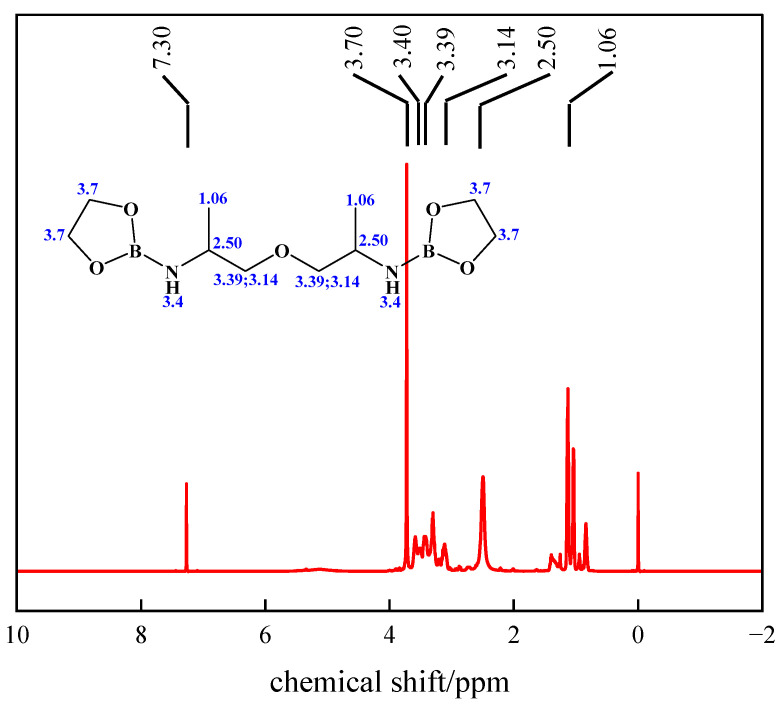
The ^1^H NMR of PBC.

The molecular structure of PBC was further elucidated by ^1^H NMR spectroscopy, with the spectrum shown in Figure 2. As shown in Figure 2, the single or broad peak at chemical shift δ = 1.06 ppm corresponded to the methyl group (-CH_3_). The peak at δ = 2.50 ppm originated from the methylene group adjacent to the nitrogen atom (-CH-NH-). The peak at δ = 3.14 ppm corresponded to the methylene group adjacent to the oxygen atom (-CH_2_-NH-). The multiple signals appearing in the δ = 3.39–3.40 ppm region primarily corresponded to the methylene group (-CH_2_-O-) bonded to the oxygen atom and N-H, originating from the ether bond units in the polyetheramine chain segments. This peak group may exhibit splitting due to the influence of adjacent chiral centers or ring system conformations. The peak at δ = 3.70 ppm was likely attributable to the methylene proton (-CH_2_-O-B-) closely adjacent to the boron-oxygen ring or ether bond, whose relatively low-field shift was influenced by the electron-withdrawing effect of the boron atom [29]. These characteristic signals collectively confirm that the hydrogen environments in the synthesized product are in good agreement with the expected structure of the target compound.

### 2.3. Univariate Analysis

**Figure 3:**
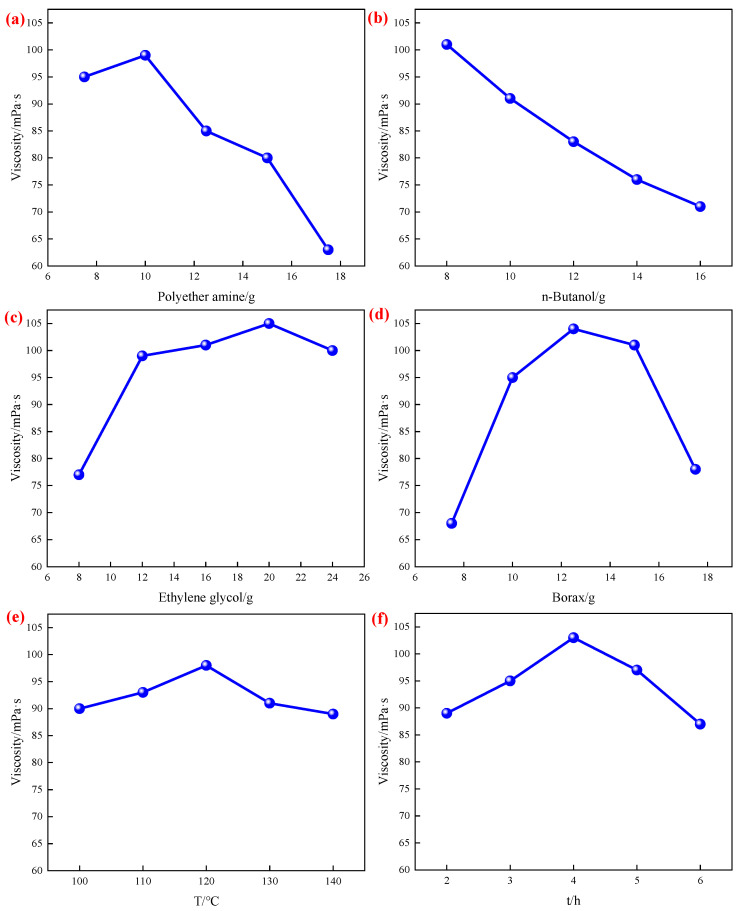
The effect of reaction conditions on viscosity ((**a**) is the effect of polyetheramine on viscosity, (**b**) is the effect of n-butanol on viscosity, (**c**) is the effect of ethylene glycol on viscosity, (**d**) is the effect of boric acid on viscosity, (**e**) is the effect of reaction temperature on viscosity, (**f**) is the effect of reaction time on viscosity).

The viscosity of crosslinked gel fracturing fluid is closely related to the synthesis conditions of PBC. The synthesis of PBC is mainly influenced by polyetheramine, ethylene glycol, n-butanol, boric acid, reaction time, and reaction temperature. Therefore, it is necessary to determine the degree of influence of each factor through single factor analysis, as shown in Figure 3.

Figure 3a shows the effect of polyetheramine on the viscosity of crosslinked gel fracturing fluids. It can be seen from Figure 3a that the amount of polyetheramine increased from 7.5 g to 17.5 g, and the viscosity of crosslinked gel initially increased slightly and then decreased steadily. The peak viscosity (99 mPa·s) was reached at 10 g. This indicated that an appropriate amount of polyetheramine promoted the formation of an effective crosslinked network, but excessive amounts may increase steric hindrance between molecular chains or lead to an excess of amino groups in the reaction system. This affected the crosslinking efficiency of boric acid, thereby causing the viscosity to decrease.

Figure 3b shows the effect of n-butanol on the viscosity of crosslinked gel fracturing fluid. It can be seen from Figure 3b that the addition of n-butanol increased from 8 g to 16 g, and the viscosity gradually decreased from 101 mPa·s to 71 mPa·s. This indicated that n-butanol, acting as a reaction medium or cosolvent, diluted the reaction system when its amount increased. This reduction in the effective concentration of reactants may lead to decreased crosslinking density, thereby lowering viscosity.

Figure 3c shows the effect of ethylene glycol on the viscosity of crosslinked gel fracturing fluid. It can be seen from Figure 3c that the addition of ethylene glycol increased from 8 g to 24 g, and the viscosity gradually increased to a maximum of 20 g (105 mPa·s) before decreasing. This indicated that ethylene glycol may function as an auxiliary crosslinker or structural modifier. At optimal concentrations, it enhanced molecular chain flexibility and crosslinking uniformity. However, excessive amounts may introduce too many hydroxyl groups, competing for boron binding sites and slightly weakening the primary crosslinking effect.

Figure 3d shows the effect of boric acid on the viscosity of crosslinked gel fracturing fluid. It can be seen from Figure 3d that within the range of 7.5 g to 17.5 g of boric acid dosage, the viscosity showed a trend of first increasing and then decreasing, reaching a peak at 12.5 g (104 mPa·s). The results indicated that boric acid, as the core component of the crosslinker, directly influenced the degree of crosslinking between boric acid and the amino groups in polyetheramine based on its dosage. An appropriate amount of boric acid formed a stable three-dimensional network structure, whereas excessive amounts may lead to overly dense crosslinking points or localized agglomeration. This, in turn, compromises the overall structural uniformity and viscosity maintenance.

Figure 3e shows the effect of reaction temperature on the viscosity of crosslinked gel fracturing fluid. It can be seen from Figure 3e that the highest viscosity (98 mPa·s) occurred at 120 °C within the reaction temperature range of 100 °C to 140 °C. The results indicated that the reaction was incomplete at excessively low temperatures. Conversely, excessively high temperatures may intensify side reactions or cause decomposition of certain components, thereby compromising the stability of the crosslinked structure.

Figure 3f shows the effect of reaction time on the viscosity of crosslinked gel fracturing fluid. It can be seen from Figure 3f that the reaction time ranged from 2 h to 6 h, and the viscosity reached its highest value (103 mPa·s) at 4 h, followed by a decrease. This indicated that 4 h may be the optimal reaction time for this system. A shorter duration may result in incomplete reaction, while a longer duration may lead to aging or degradation of the crosslinked structure.

Based on the single-factor experimental analysis, polyetheramine, n-butanol, and ethylene glycol exhibited the most significant influence on the viscosity of the crosslinked gel. Specifically, in terms of data, these three components caused the largest variation in viscosity and showed the most pronounced trends. For instance, an increase in the amount of n-butanol led to a substantial linear decrease in viscosity, whereas ethylene glycol, at an optimal dosage, resulted in the highest viscosity value within the system. Mechanistically, they directly and sensitively governed the formation of the three-dimensional cross-linked network from three key aspects: The construction of the main structure of the cross-linked network (polyetheramine), the regulation of the reaction microenvironment and concentration (n-butanol), and the competition in cross-linking along with the modification of the network microstructure (ethylene glycol). Therefore, for the response surface design, these three were identified as core variables to be prioritized for further investigation and precise control.

### 2.4. Variance Analysis and Regression Model

The relationship between reaction conditions and viscosity was analyzed using the Box–Behnken module. A multivariate fitting regression equation was established. The second-order response surface equation is shown in Equation (1). In the equation, Y represents viscosity, *X*_1_ denotes the ethylene glycol dosage, *X*_2_ indicates the n-butanol dosage, and *X*_3_ signifies the polyetheramine dosage.

(1) $$\begin{array}{l} Y=101.2+2.37X_{1}-3.25X_{2}+7.13X_{3}+2.25X_{1}X_{2}+0.5X_{1}X_{3}-\\ 4.75X_{2}X_{3}-22.6X_{1}^{2}-11.35X_{2}^{2}-18.1X_{3}^{2} \end{array}$$

Typically, the accuracy of models established using response surface methodology can be tested through *p*-values and F-values in statistical analysis. If the significance test of the regression model shows that the *p*-value is less than 0.05, or the F-regression value is greater than the critical value F_0.05_. This indicates that the model has high significance, and the correlation between the dependent variable and the independent variable is significant at a 95% confidence level. If the *p*-value is less than 0.01 or the F-regression value is greater than F_0.01_. This indicates that the model is extremely significant, with a confidence level of up to 99% [30]. Based on the above statistical criteria, this study established a second-order response surface model between viscosity and reaction conditions, and the corresponding statistical analysis results are shown in Table 1.

Table 1 shows that the F-value of the regression model was 285.29, which was greater than F_0.01_(9,4) = 14.66, and the *p* value was less than 0.0001. This indicated that the established second-order multiple regression model was highly significant. The F-value for the misfit term was 2.57, which was less than F_0.05_(9,3) = 8.81, and the *p*-value for the misfit term was 0.1921 (>0.05). This indicated that the misfit of the established second-order multiple regression model was not significant, and the regression equation exhibited a high degree of fit. Additionally, the *p*-values for the linear terms *X*_1_, *X*_2_, *X*_3_ and the quadratic terms *X*_1_^2^, *X*_2_^2^, *X*_3_^2^ were all less than 0.05. This indicated that the effects of these terms on the response value were all statistically significant. Furthermore, based on the magnitude of the F-values for the linear terms, the order of significance for the influence of each reaction condition on viscosity was determined to be polyetheramine > n-butanol > ethylene glycol. The model results showed that the coefficient of determination R^2^ and the adjusted coefficient of determination R^2^_adj_ were 0.9973 and 0.9938, respectively. This indicated that the model had a high degree of fit and could be used to optimize and predict the synthesis process conditions for PBC. The significant interaction effects revealed in our analysis—particularly the synergistic interplay between polyetheramine, ethylene glycol, and n-butanol—provide scientific insights beyond simple optimization. The observation that polyetheramine exerts the most dominant influence on viscosity (F = 202.34, *p* < 0.0001), and that its interaction with n-butanol is highly significant (F = 44.96, *p* = 0.0003), suggests that the amino-containing flexible segments play a critical role in network formation, and that the solvent environment during synthesis modulates this effect.

**Figure 4:**
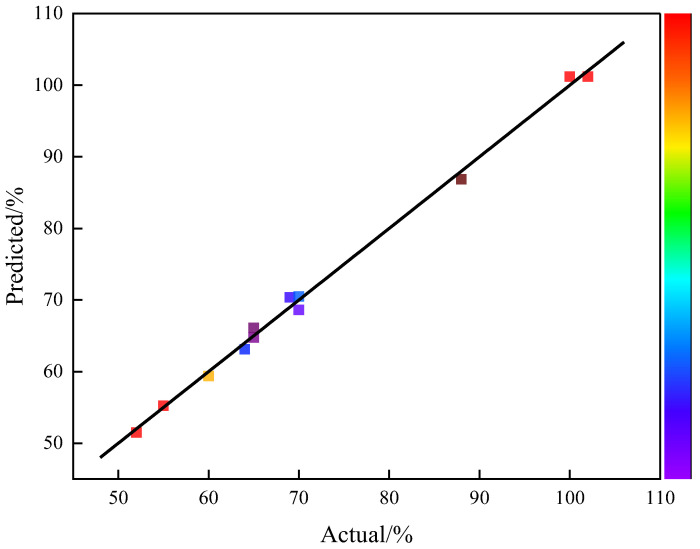
Relationship between actual and predicted values.

The satisfaction of the second-order response surface model can be evaluated by the normal distribution of residuals, as shown in Figure 4. This graph illustrates the relationship between actual viscosity and predicted viscosity. It can be seen that all data points were closely distributed near the fitting line. This indicated that the model was established reasonably and the predicted results had high credibility.

### 2.5. Interaction Between Variable Factors

**Figure 5:**
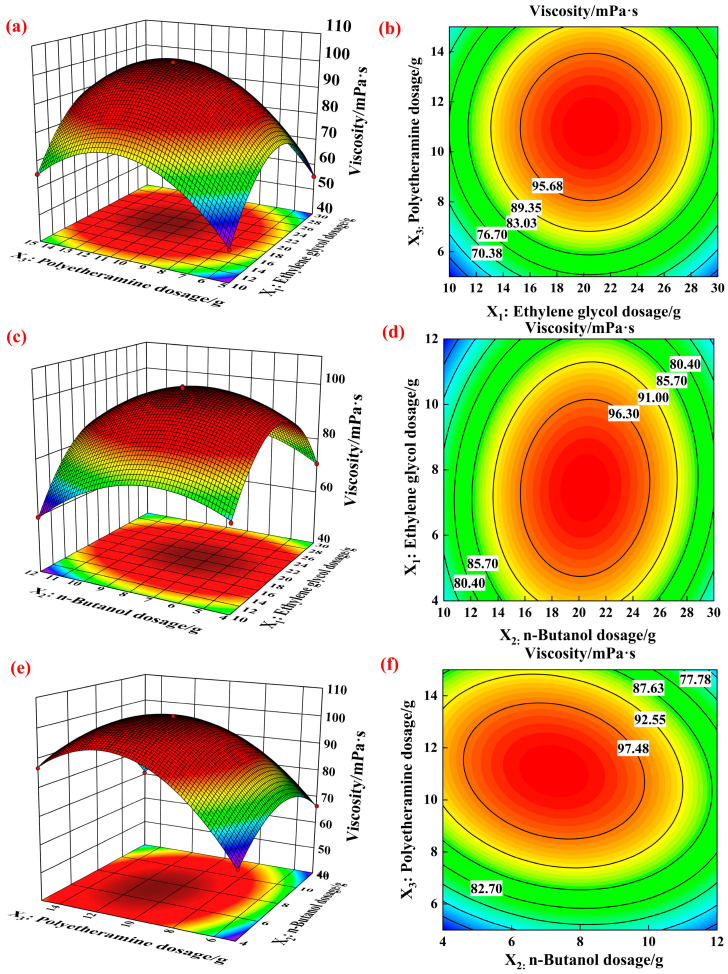
3D surface and contour plots of reaction conditions and viscosity ((**a**,**b**) are 3D surface and contour plots of the effects of ethylene glycol and polyetheramine on viscosity. (**c**,**d**) are 3D surface and contour plots of the effects of ethylene glycol and n-butanol on viscosity. (**e**,**f**) are 3D surface and contour plots of the effects of polyetheramine and n-butanol on viscosity).

Through the second-order response surface model, it can be found that polyetheramine, n-butanol, and ethylene glycol had a significant impact on viscosity. Therefore, in order to more accurately reflect the influence of process conditions on viscosity, the interaction between various factors was studied using response surface methodology. The experimental results are shown in Figure 5.

Figure 5a,b show the 3D surface and contour plots of the effects of ethylene glycol and polyetheramine on viscosity under a certain amount of n-butanol addition. It can be seen from Figure 5a that as the amount of ethylene glycol and polyetheramine increased, the viscosity first increased and then decreased. The results showed that the viscosity reached its maximum when the amount of ethylene glycol was between 20.23–20.89 g and the amount of polyetheramine was between 10.32–10.96 g. It can be seen from Figure 5b that the contour lines towards the direction of polyetheramine were denser than those towards the direction of ethylene glycol, and the contour lines were elliptical in shape. The results indicated that polyetheramine exerted a greater influence on viscosity than ethylene glycol, and the interaction between these two factors significantly affected viscosity. This suggested that polyetheramine was more sensitive to viscosity changes in this region. This may be attributed to the controlled cross-linking between the amine groups in polyetheramine molecules and borate ester bonds, which enhanced the network structure within a certain range. However, excessive amounts may induce steric hindrance or localized crosslinking irregularities, which could actually hinder overall viscosity improvement.

Figure 5c,d show the 3D surface and contour plots of the effects of ethylene glycol and n-butanol on viscosity at a certain amount of polyetheramine addition. It can be seen from Figure 5c that as the amount of ethylene glycol and n-butanol increased, the viscosity first increased and then decreased. The results showed that the viscosity reached its maximum when the amount of ethylene glycol was between 19.98–20.89 g and the amount of n-butanol was between 7.01–8.12 g. It can be seen from Figure 5d that the contour lines towards n-butanol were denser than those towards ethylene glycol, and the contour lines were elliptical in shape. The results indicated that n-butanol had a greater impact on viscosity than ethylene glycol, and the interaction between the two factors had a more significant effect on viscosity. The two had a synergistic effect in forming effective cross-linked structures, and excessive or insufficient cross-linking could lead to a decrease in cross-linking density.

Figure 5e,f show the 3D surface and contour plots of the effects of polyetheramine and n-butanol on viscosity at a certain amount of ethylene glycol. It can be seen from Figure 5e that as the amount of polyetheramine and n-butanol increased, the viscosity first increased and then decreased. The results showed that the viscosity reached its maximum when the dosage of polyetheramine was between 10.42–10.86 g and the dosage of n-butanol was between 7.11–7.98 g. It can be seen from Figure 5f that the contour lines facing the direction of polyetheramine were denser than those facing the direction of n-butanol, and the contour lines were elliptical in shape. The results indicated that polyetheramine had a greater impact on viscosity than n-butanol, and the interaction between the two factors had a more significant effect on viscosity. This indicated that n-butanol played a role in regulating the polarity and solubility of the reaction system. An appropriate amount of n-butanol facilitated the dispersion of polyetheramine and promoted cross-linking reactions. However, excessive amounts may dilute the reaction system, thereby reducing the density of effective cross-linking sites.

**Figure 6:**
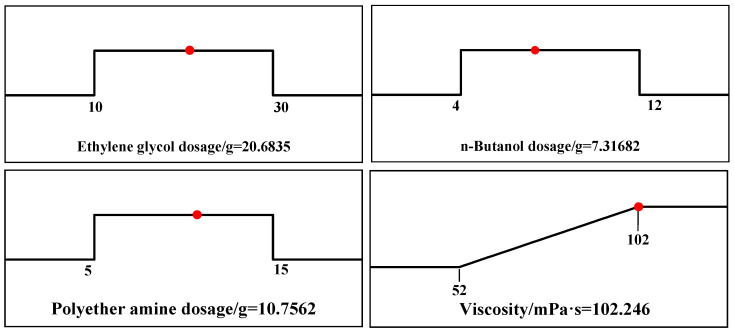
Optimal factor analysis and viscosity prediction plot.

Figure 6 shows the optimal synthesis conditions obtained from the Box–Behnken model. The predicted viscosity is 102.246 mPa·s for polyetheramine at 10.7562 g, n-butanol at 7.31682 g, and ethylene glycol at 20.6835 g. To verify the error between the predicted yield and the actual yield. Validation was conducted under reaction conditions of 10.8 g of polyetheramine, 7.4 g of n-butanol, and 20.7 g of ethylene glycol. The actual viscosity was 101.5 mPa·s, with a relative error of 0.7%. The error was relatively small, and the accuracy of the model parameters established by this method had a certain reference value.

Based on the above analysis, polyetheramine had the most significant impact on viscosity, and the polyamine groups in its molecular structure were the core of forming a cross-linked network. Ethylene glycol and n-butanol synergistically affected the crosslinking process and network uniformity by adjusting the reaction activity and solvent environment of the system. This study not only clarified the primary and secondary relationships of various factors through response surface methodology, but also revealed the complex nonlinear interaction mechanism among the three. This provides theoretical basis and experimental guidance for precise optimization of the PBC synthesis process and regulation of fracturing fluid performance in the future.

### 2.6. Temperature and Shear Resistance Performance

**Figure 7:**
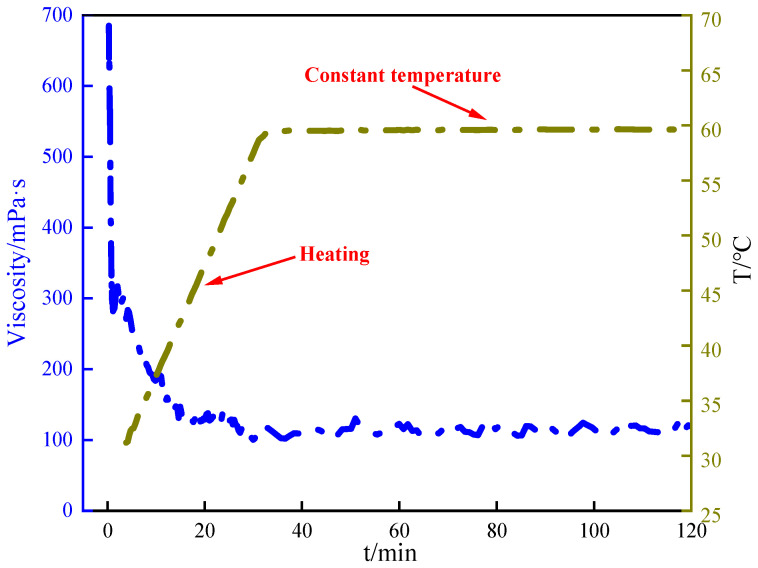
Temperature resistance and shear resistance of crosslinked gel fracturing fluid.

Temperature and shear resistance testing is a key means of evaluating the viscosity stability of fracturing fluid at a certain temperature and shear rate, which directly affects the sand-carrying capacity and fracturing effect of fracturing fluid in complex underground environments. Therefore, it is necessary to test the temperature resistance and shear resistance of crosslinked gel fracturing fluid, as shown in Figure 7. As shown in Figure 7, the viscosity of the crosslinked gel fracturing fluid generally decreased initially and then gradually stabilized with increasing temperature and shear time. The higher viscosity in the initial stage may be attributed to the formation of a spatial network structure between PBC and guar gum. Subsequently, under sustained heating and high shear stress, polymer chains may undergo chain breakage or disruption of crosslinking points, partially destroying the network structure and thereby reducing viscosity. The viscosity of crosslinked gel fracturing fluid decreased to about 100 mPa·s and tended to be stable. This trend indicated that PBC maintained good structural stability within certain temperature and shear time ranges, closely related to the heat resistance and shear sensitivity of its chemical bonds. This behavior demonstrated the elasticity and thermal adaptability of crosslinked gel fracturing fluids, which were critical for hydraulic fracturing in deep or high-temperature unconventional reservoirs [31,32].

### 2.7. Static Filtration Performance

**Figure 8:**
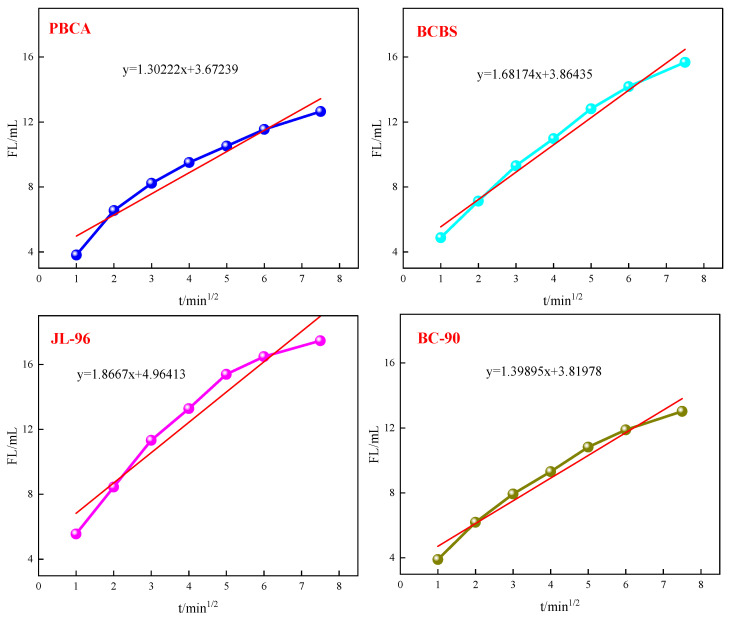
Static filtration curve of crosslinked gel fracturing fluid.

Static filtration performance testing is a key indicator for evaluating the filtration control ability of fracturing fluid under reservoir temperature and pressure difference conditions. It is directly related to the efficiency of filter cake formation and liquid efficiency during the fracturing process, which in turn affects the fracturing fluid’s fracturing and sand-carrying performance. The static loss curve of the crosslinked gel fracturing fluid is shown in Figure 8. The regression equation fitted from the static loss curve is presented in Table 2.

It can be seen from Table 2 that the wall forming filtration coefficient of the gel fracturing fluid prepared with PBC was 2.3 × 10^−4^ m/s^½^, and the initial filtration was 1.30 × 10^−3^ m^3^/m^2^. It demonstrated outstanding performance, with values significantly lower than those of the comparative crosslinkers BCBS, JL-96, and BC-90. The results indicated that the PBC crosslinking system formed a dense and stable filter cake more rapidly under static high-temperature conditions, thereby effectively reducing filtrate loss into the formation. This performance difference likely stemmed from the higher crosslinking efficiency between functional groups in the PBC molecular structure and polymer chains. This enabled the formation of a more uniform and dense spatial structure within the gel network, enhancing the confinement of free water and improving the compaction and low permeability of the filter cake [33,34]. Consequently, PBC was suitable for high-temperature reservoir fracturing operations with stringent loss control requirements, contributing to enhanced fracturing fluid efficiency and proppant distribution.

### 2.8. Sand-Carrying Performance

**Figure 9:**
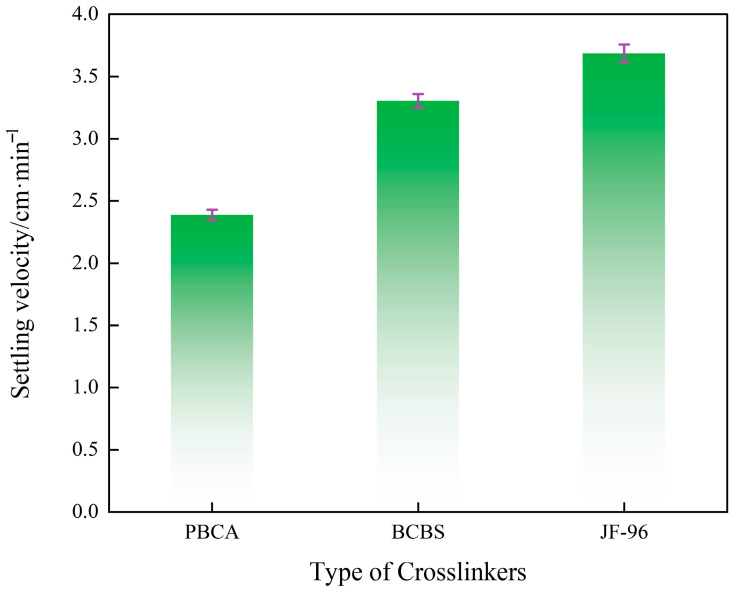
The effect of different crosslinkers on the settling velocity of quartz sand in gel fracturing fluids.

Static proppant suspension testing is a critical experimental method for evaluating the suspension and proppant-carrying capacity of fracturing fluids under static conditions. It directly impacts the uniformity of proppant distribution within fractures and the final sand packing effect during fracturing, playing a vital role in ensuring the flow capacity of fractures post-fracturing. Figure 9 presents the results of this test, illustrating the effect of different crosslinker types on the settling velocity of 20–40 mesh quartz sand in a 0.2% guar gum fracturing fluid at 60 °C. For all samples, the crosslinker concentration was kept constant at 0.4% (*v*/*v*), and the sand-to-fluid ratio was maintained at 30% (*w*/*v*). It can be seen from Figure 9 that the settling velocity of quartz sand in the fracturing fluid prepared by PBC was 2.4 cm·min^−1^, which was lower than BCBS (3.3 cm·min^−1^) and JL-96 (3.7 cm·min^−1^), indicating that the PBC system had better static suspension ability. The results showed that the crosslinked gel network structure formed by PBC was more uniform and stable, and could more effectively hinder the sedimentation of proppant particles. The results showed that the crosslinked gel network structure formed by PBC was more uniform and stable, and could more effectively hinder the sedimentation of proppant particles. This may be attributed to the interaction between specific functional groups in PBC molecules and the guar gum fracturing fluid base, which can form a three-dimensional network structure with better spatial extensibility and stronger elasticity [35,36]. Thus, the overall viscoelasticity and cohesion of gel can be enhanced, and its ability to wrap and support solid particles can be improved. Therefore, in actual fracturing construction, the use of the PBC cross-linking system can help improve the retention and distribution effect of proppants in fractures, thereby enhancing the fracture conductivity and fracturing transformation efficiency.

### 2.9. The Content of Residue After Gel Breakage

**Figure 10:**
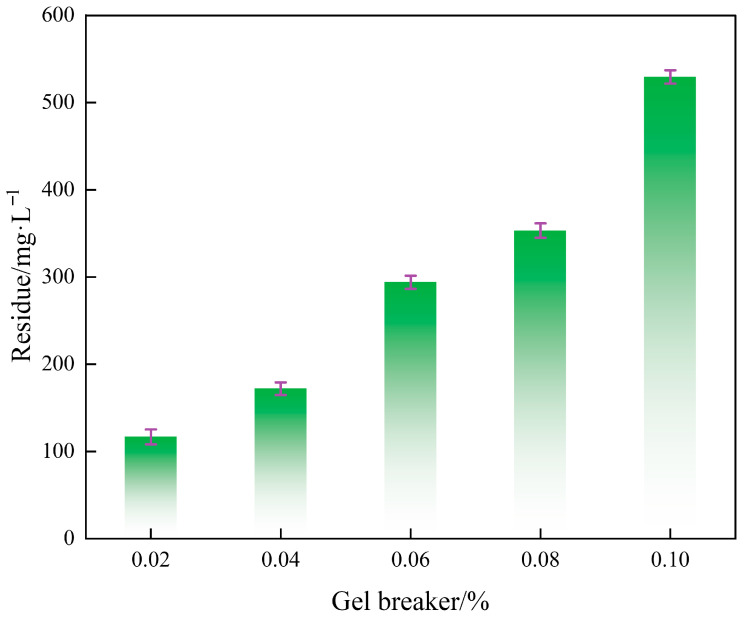
Effect of gel breaker concentration on the residue content of crosslinked gel fracturing fluid after complete gel breaking.

The determination of residual content after fracturing is a key test for evaluating the thoroughness of fracturing fluid and the potential for reservoir damage, which directly affects the fracture conductivity and reservoir protection effect after fracturing. As shown in Figure 10, in crosslinked gel fracturing fluids, as the dosage of gel breaker gradually increased from 0.02% to 0.1%, the residual content correspondingly rose continuously from 117 mg/L to 531 mg/L. The results showed that increasing the dosage of gel breaker failed to degrade the residue and instead led to an increase in residue content. This phenomenon can be attributed to two main factors. On the one hand, ammonium persulfate (APS), as an oxidative breaker, may cause excessive oxidation and breakage of guar gum molecular chains at higher concentrations. This would generate more insoluble substances that were difficult to dissolve or aggregate [37]. On the other hand, the cross-linked structure in gel may be degraded unevenly under the action of excessive breaker. Some retained or reconstructed cross-linking points were left to form stable micro gel particles, which were then captured as residues during centrifugation [38]. Therefore, although all measured residue levels remained below the conventional specification limit of 600 mg/L, the optimization of gel breaker dosage must balance breaking speed with residue control. Excessive pursuit of rapid breaking may abnormally increase residue content, thereby impairing reservoir permeability and fracture conductivity. These insights contribute to the optimization of fracturing fluid formulations, the reduction of formation damage, and the improvement of fracture conductivity and stimulation effectiveness, offering important guidance for achieving efficient and low-damage volume fracturing.

### 2.10. Mechanism Analysis

The mechanism of action of PBC crosslinker primarily stemmed from their unique molecular structure and the stable, dynamically adaptive gel network formed through synergistic multiple interactions. The PBC molecule integrates borate ester structures and polyetheramine segments, which may potentially enable B–N coordination interactions. Although such interactions could theoretically enhance the stability and dynamic behavior of the crosslinked network, it is important to emphasize that this remains a hypothesis in the current study. Direct spectroscopic evidence, such as ^11^B NMR or X-ray photoelectron spectroscopy (XPS), would be required to confirm the presence and nature of B–N bonding. This enabled it to exhibit multiple bonding capabilities when crosslinking with polymer solutions such as guar polymers [39]. Here, boron atoms served as the crosslinking core, forming reversible coordination bonds with cis-hydroxy groups (-OH) on guar polymer chains to construct a three-dimensional network framework with dynamic properties. This dynamic crosslinking not only conferred the gel with favorable initial viscosity and elasticity but also enabled energy dissipation through bond breaking and reformation under high-temperature shear [40,41]. The dynamic reversible crosslinking in the PBC-guar system originates from the inherent reversibility of boronic ester bonds, which can undergo exchange and recombination under shear and thermal conditions. This dynamic behavior is further facilitated by the flexible polyetheramine segments, which provide chain mobility and enable energy dissipation through reversible bond cleavage without permanent network damage. The stable viscosity plateau observed under continuous high shear (Figure 7) serves as macroscopic evidence of this dynamic equilibrium, where the rates of bond rupture and reformation are balanced, maintaining overall network integrity. Consequently, it demonstrated outstanding thermal and shear resistance.

Meanwhile, the flexible spacers provided by the polyetheramine segments in PBC, along with ether oxygen atoms (C-O-C), enhanced the molecular chain’s flexibility and hydration capacity [42]. This facilitated the formation of a uniform and dense gel network. Such a network effectively trapped free water and reduced the connectivity of filtration pathways. Consequently, it significantly lowered the wall-forming filtration coefficient and initial filtration, thereby improving filtration control performance. Furthermore, the highly crosslinked and elastically resilient network structure provided effective spatial confinement and physical encapsulation of proppant particles, slowing their settling velocity. This manifested as a lower static settling rate, indicating superior sand suspension capability.

During gel breakdown, moderate crosslinking density and dynamic bonding characteristics may cause the gel to preferentially generate smaller, easily dispersible fragments during network degradation [43]. However, excessive gel breakers may induce localized excessive network breakage and rearrangement into stable microgels, thereby increasing residue content [44]. This interpretation is consistent with the residue data presented in Figure 10. Therefore, the comprehensive performance advantages of PBC can be attributed to the synergistic effect of its “rigidity-flexibility” balanced molecular design and multiple dynamic crosslinking mechanisms. This enables it to provide stable, controllable rheological properties and reservoir protection for fracturing fluids under multiple harsh conditions, including high temperatures, shear stress, and fluid loss.

## 3. Conclusions

This study successfully designed and synthesized a novel polyamine boron crosslinker (PBC) for low-concentration (0.2%) guar gum fracturing fluid system. A highly significant second-order regression model was established through one-pot synthesis and optimization of raw material ratios using response surface methodology. The optimal synthesis conditions were determined to be 10.8 g of polyetheramine, 7.4 g of n-butanol, and 20.7 g of ethylene glycol, with a prediction error of only 0.7%. The structural characterization results (FT-IR and ^1^H NMR) confirmed that PBC molecules contained expected boronic ester bonds and polyetheramine segments, providing molecular-level evidence for their performance. The results indicated that PBC exhibited significant advantages. The viscosity of crosslinked gel fracturing fluid was kept at approximately 100 mPa·s under 60 °C and 100 s^−1^ shear, demonstrating excellent thermal shear resistance. The filtration control ability was outstanding, with a wall forming filtration coefficient of 2.30 × 10^−4^ m/s^1/2^ and an initial filtration of 1.30 × 10^−3^ m^3^/m^2^, both lower than those of the comparative commercial crosslinker. Mechanistic analysis indicated that the synergistic interaction between boron ester bonds and polyetheramine segments in PBC conferred dynamic reversible crosslinking properties and a uniform three-dimensional network structure. This enabled high-strength, low-damage crosslinking effects at low thickener concentrations.

This study provides an efficient and controllable crosslinker solution for low-concentration guar gum fracturing fluids, offering both a theoretical basis for the molecular design of organic boron crosslinkers and a methodological reference for synthesis process optimization, thereby contributing to the development of greener, more cost-effective, and higher-performance fracturing fluid systems. However, several limitations should be acknowledged. The thermal stability evaluation was conducted only at 60 °C for 120 min, and long-term stability over extended periods under static conditions requires further investigation. Additionally, the proposed B–N coordination mechanism remains hypothetical and requires validation through advanced spectroscopic techniques such as ^11^B NMR or XPS. Furthermore, while the PBC system demonstrated superior performance compared to commercial crosslinkers, direct assessment of formation damage through core flooding experiments would provide more definitive evidence of its low-damage characteristics. To address these limitations, future research directions include evaluating the PBC system at higher temperatures (>80 °C) to expand its application range, conducting oscillatory rheological recovery tests to quantitatively assess dynamic reversible crosslinking behavior, exploring additional polyamine structures to further optimize molecular design, and validating system performance under dynamic well conditions through pilot field trials.

## 4. Materials and Methods

### 4.1. Materials and Reagents

Boric acid (analytical grade) and ethylene glycol (analytical grade) were purchased from Beijing Xinbaohai Chemical Technology Co., Ltd., Beijing, China. N-butanol (analytical grade) and polyetheramine (analytical grade) were purchased from Shanghai McLean Biochemical Technology Co., Ltd., Shanghai, China. Quartz sand was purchased from Shanghai Aladdin Biochemical Technology Co., Ltd., Shanghai, China. The clay stabilizer TAP-1, ZP-2, crosslinker JL-90, and regulator SJ-3 were taken from Tianjin Tiancheng Tuoyuan Technology Development Co., Ltd., Tianjin, China. The fungicide TYJ-2, crosslinker BCBS, and crosslinker BC-90 were taken from Beijing Shida Bocheng Technology Co., Ltd., Beijing, China. Fourier transform infrared (FTIR) spectra were recorded on a Nicolet Magna-560 FT-IR spectrometer (Nicolet, Madison, WI, USA) using KBr pellets. The NMR spectrometer used in this study is a Bruker AVANCE III 600 MHz (Bruker, Billerica, MA, USA). Design-Expert software is the 13th edition, Stat-Ease, Inc., Minneapolis, MN, USA.

### 4.2. Synthesis of PBC

**Figure 11:**
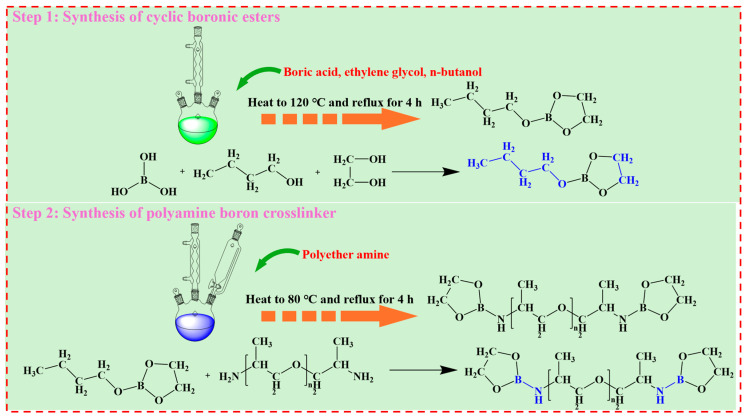
Schematic diagram of the PBC synthesis process.

This experiment employed a one-pot synthesis method to produce the target polyamine boron crosslinker, and the reaction process is shown in Figure 11. The specific procedure is as follows: Precisely weighed boric acid, ethylene glycol, and n-butanol were sequentially added to a three-neck round-bottom flask equipped with a magnetic stir bar, thermometer, and reflux condenser. Stirring was initiated, the cooling water circulation system was activated, and the reaction apparatus was placed in an oil bath preheated at 120 °C to form borate ester intermediates. Under continuous stirring and reflux cooling conditions, the reaction mixture gradually reached the target temperature and was maintained at this temperature for 4 h to ensure complete reaction. Subsequently, upon addition of polyetheramine at 80 °C, the amino groups react with or coordinate to the borate centers. After the reaction, the flask was removed from the oil bath and allowed to cool to near room temperature at ambient conditions, yielding the crude product. To purify the target product, the crude product was transferred to a round-bottom flask. Excess low-boiling-point components and reaction-generated water were removed under reduced pressure at 40 °C using a rotary evaporator. The resulting viscous liquid was dissolved in an appropriate amount of anhydrous ethanol and precipitated using an appropriate amount of n-hexane. The precipitate was collected by vacuum filtration and washed twice with a small amount of cold n-hexane to further remove residual impurities. The final product was dried in a vacuum oven at 40 °C to constant weight, yielding a light yellow to amber viscous liquid polyamine boron crosslinker, denoted as PBC.

### 4.3. Optimized Design

The main influencing factors of the reaction were determined through univariate analysis. Subsequently, response surface methodology was used to design optimization experiments by Design-Expert software (Version 13, Stat-Ease, Inc., Minneapolis, MN, USA). Finally, determine the optimal conditions for the reaction. The three influencing factors identified in this study are ethylene glycol, n-butanol, and polyetheramine. In addition, the third-level variables are encoded as −1, 0, and 1, as shown in Table 3.

The optimized experimental scheme and viscosity for the synthesis reaction conditions of PBC are shown in Table 4. Design a second-order polynomial response equation using Box–Behnken to analyze the relationships between variables [45]. Where *Y* is the value of the dependent variable, *β*_0_ is the regression constant, the independent variables are represented by *X*_i_ and *X*_j_, and *β*_i_, *β*_i_ and *β*_ij_ represent the first-order linear regression coefficients, the quadratic regression coefficients, and the interaction effect coefficients, respectively.

(2) $$Y=\beta_{0}+\sum_{i=1}^{K}\beta_{i}X_{i}+\sum_{i=1}^{K}\beta_{ii}X_{i}^{2}+\sum_{i=1}^{K}\sum_{i\neq j}^{K}\beta_{ij}X_{i}X_{j}$$

### 4.4. Characterization of PBC

The chemical structure of the synthesized product was characterized using a MAGNA-560 Fourier Transform Infrared Spectrometer (FT-IR). Its molecular configuration was confirmed by analyzing characteristic functional groups [46]. Testing was conducted in transmission mode, with the spectral scan range spanning wavelengths from 400 to 4000 cm^−1^. The molecular structure of the PBC sample was characterized by ^1^H NMR spectroscopy, which was performed on a Bruker AVANCE III 600 MHz spectrometer at ambient temperature using deuterated chloroform (CDCl_3_) as the solvent [47].

### 4.5. Performance Evaluation of Crosslinking

#### 4.5.1. Preparation of Guar Gum Fracturing Fluid

A 0.2% guar gum fracturing fluid base solution was prepared as follows. First, 1000 mL of tap water was added to a beaker and stirred using a magnetic stirrer. Subsequently, 2 g of guar gum powder was introduced into the water under continuous stirring to obtain a homogeneous aqueous solution. Then, the following additives were sequentially added to the guar gum solution: 5 g of a clay stabilizer (TAP-2), 3 g of a flowback aid (ZP-2), 1 g of a regulator (SJ-3), and 1 g of a biocide (TYJ-2). After thorough mixing, the solution was transferred to a water bath and maintained at 30 °C for 4 h under constant heating. The resulting mixture was used as the 0.2% guar gum fracturing fluid base solution [48].

#### 4.5.2. Determination of Crosslinked Gel Viscosity

To prepare the crosslinked gel, 100 mL of the previously prepared guar gum base solution was placed in a beaker. A synthesized crosslinker was then added at a mass ratio of base solution to crosslinker of 100:0.4. The mixture was stirred at room temperature until a homogeneous crosslinked gel formed. The gel was subsequently placed in a constant-temperature environment at 60 °C (simulating the target reservoir temperature) and maintained for 2 h to allow for thermal maturation [49]. The viscosity of the matured crosslinked gel was measured using a six-speed rotational viscometer (Haitongda, Qingdao, China). The reported viscosity value represents the average of three independent replicate measurements.

### 4.6. Temperature and Shear Resistance Performance Testing

In practical applications, fracturing fluid systems are simultaneously subjected to high temperature and intense shear under reservoir conditions, often leading to a significant decrease in viscosity, thereby compromising fracturing effectiveness. Therefore, the thermal resistance and shear resistance of the fracturing fluid system are crucial for maintaining its performance under such complex working conditions. To evaluate the performance of the prepared cross-linked gel fracturing fluid under these conditions, its viscosity was tested using a HAAKE MARS rotational rheometer equipped with a coaxial cylinder rotor system (Thermo Fisher Scientific, Waltham, MA, USA). During the test, the temperature was first increased from the initial condition to 60 °C at a constant rate of 3.0 °C/min, and then maintained at this temperature while applying a constant shear rate of 100 s^−1^ for 120 min [50].

### 4.7. Static Filtration Performance Testing

The prepared crosslinked gel was maintained at 60 °C for 16 h and then allowed to cool to room temperature. A certain amount of the cooled crosslinked gel was placed into a fluid loss cell, and the system pressure was adjusted to 0.69 MPa via a pressure-reducing valve. The bottom valve was then opened, and the filtrate was collected using a graduated cylinder from the moment the first drop emerged. The cumulative filtrate volumes were recorded at 1 min, 2 min, 3 min, 4 min, 5 min, 6 min, and 7.5 min. According to the relationship between the fluid loss coefficient and the square root of time specified in SY/T7627-2021 “General Specifications for Fracturing Fluids,” linear regression was performed on the obtained data points to determine the intercept *B* and slope *M* of the regression line. Using the slope and intercept, the wall-forming fluid loss coefficient *C*_w_ and the initial filtration *S*_L_ can be calculated using Equations (3) and (4), respectively. Where *C*_w_ is the wall filtration coefficient (m/s^½^). *S*_L_ is the initial filtering vector (m^3^/m^2^). A is the cross-sectional area of the filter medium (m^2^). *B* is the intercept (m^3^). *M* is the slope (m^3^/s^½^).

(3) $$C_{W}=M/2A$$

(4) $$S_{L}=B/A$$

### 4.8. Sand-Carrying Performance Testing

The static proppant suspension test was conducted following the API standard procedure [51]. The prepared gel fracturing fluid system was first poured into a 100 mL graduated cylinder and allowed to reach thermal equilibrium at the specified test temperature. Subsequently, 20–40 mesh quartz sand was added to the cylinder at a sand-to-fluid ratio of 30% (*w*/*v*). The mixture was stirred thoroughly to ensure uniform dispersion of the sand within the gel. The time required for the sand particles to settle completely to the bottom of the cylinder was recorded. Based on this settling time and the known fluid column height, the free settling velocity of the sand particles was calculated [52]. The experimental measurements were performed in triplicate and the data are presented as mean values.

### 4.9. Determination of Residue Content After Gel Breakage

The residue content in the thoroughly broken fluid (with no re-gelling observed) from the aforementioned experiment was determined using the gravimetric method [53]. Specifically, eight 50 mL centrifuge tubes were cleaned, dried to constant weight, and weighed (recorded as *M*_1_). The broken fluid was then poured into the tubes and centrifuged at 3000 rpm for 30 min. After discarding the supernatant, the precipitate was washed once with distilled water and centrifuged again for 25 min. The supernatant from the washing step was removed, and the tubes were placed in an oven at 80 °C until a constant weight was achieved (recorded as *M*_2_). The residue mass (Δ*M*) was calculated as Δ*M* = *M*_2_ − *M*_1_. The residue content of the fracturing fluid was calculated using Equation (5). where *η* is the residue content of the fracturing fluid (mg/L). Δ*M* is the residue mass (mg). *V* is the volume of the fracturing fluid sample (mL). In this experiment, *V* was 50 mL. The experimental measurements were performed in triplicate and the data are presented as mean values. Since residue can impair fracture conductivity, the residue content of the fracturing fluid is generally required to be ≤600 mg/L.

(5) η=(ΔM/V)×1000

## Figures and Tables

**Table 1:** Statistical analysis of second-order response surface models.

Source	Sum of Squares	Free Degree	Mean Square	F-Value	*p*-Value
Model	5153.48	9	572.61	285.29	<0.0001
X_1_	45.13	1	45.13	22.48	0.0021
X_2_	84.50	1	84.50	42.10	0.0003
X_3_	406.13	1	406.13	202.34	<0.0001
X_1_X_2_	20.25	1	20.25	10.09	0.0156
X_1_X_3_	1.0000	1	1.0000	0.4982	0.5031
X_2_X_3_	90.25	1	90.25	44.96	0.0003
X_1_^2^	2150.57	1	2150.57	1071.46	<0.0001
X_2_^2^	542.41	1	542.41	270.24	<0.0001
X_3_^2^	1379.41	1	1379.41	687.25	<0.0001
Residual	14.05	7	2.01		
Lack of fit	9.25	3	3.08	2.57	0.1921
Pure error	4.80	4	1.20		
Total error	5167.53	16			
R^2^	0.9973				
R^2^_Adj_	0.9938				

**Table 2:** Static filtration performance of cross-linked gel fracturing fluid.

Crosslinkers	Intercept/m^3^	Slope/m^3^/s^1/2^	Cross-Sectional Area/m^2^	Wall Forming Filtration Coefficient/m/s^1/2^	Initial Filtration /m^3^/m^2^
PBC	3.67239 × 10^−6^	1.30222 × 10^−6^	2.826 × 10^−3^	2.30 × 10^−4^	1.30 × 10^−3^
BCBS	3.86435 × 10^−6^	1.68174 × 10^−6^	2.826 × 10^−3^	2.98 × 10^−4^	1.37 × 10^−3^
JL-96	4.96413 × 10^−6^	1.86670 × 10^−6^	2.826 × 10^−3^	3.30 × 10^−4^	1.76 × 10^−3^
BC-90	3.81978 × 10^−6^	1.39895 × 10^−6^	2.826 × 10^−3^	2.48 × 10^−4^	1.35 × 10^−3^

**Table 3:** Actual values and coding of independent variables.

Factor	Code	Level		
		−1	0	1
Ethylene glycol/g	X_1_	10	20	30
n-Butanol/g	X_2_	4	8	12
Polyetheramine/g	X_3_	5	10	15

**Table 4:** Box–Behnken experimental design and viscosity.

Number	Ethylene Glycol/g	n-Butanol/g	Polyetheramine/g	Viscosity/mPa·s
1	0	0	0	100
2	−1	−1	0	69
3	1	0	1	70
4	−1	1	0	60
5	0	−1	−1	64
6	0	1	1	70
7	0	1	−1	65
8	1	−1	0	70
9	0	0	0	102
10	0	0	0	102
11	0	0	0	100
12	−1	0	−1	52
13	1	0	−1	55
14	0	−1	1	88
15	1	1	0	70
16	0	0	0	102
17	−1	0	1	65

## Data Availability

The data presented in this study are openly available in the article.
